# Porous tantalum rod implantation is associated with low survival rates in patients with type C2 osteonecrosis of the femoral head but has no effect on the clinical outcome of conversion total hip arthroplasty: a retrospective study with an average 8-year follow-up

**DOI:** 10.1186/s12891-020-03860-8

**Published:** 2020-12-12

**Authors:** Mincong He, Qiushi Wei, Zhenqiu Chen, Fan Yang, Xiaojun Chen, Yi-Xian Qin, Bin Fang, Wei He

**Affiliations:** 1 Department of Orthopedic Oncology, The First Affiliated Hospital of Guangzhou University of Chinese Medicine, Guangzhou University of Chinese Medicine, Guangzhou, People’s Republic of China; 2 Laboratory of Orthopaedics & Traumatology, Lingnan Medical Research Center, Guangzhou University of Chinese Medicine, Guangzhou, China; 3 Department of Biomedical Engineering, Stony Brook University, Stony Brook, NY USA; 4 Institute of Orthopedics of Guangzhou University of Chinese Medicine, Guangzhou, Guangdong China; 5 The Third Affiliated Hospital of Guangzhou University of Chinese Medicine, NO.12 Jichang Road, Guangzhou, Guangdong 510405 People’s Republic of China; 6 The First Clinical Medical College, Guangzhou University of Chinese Medicine, Guangzhou, People’s Republic of China

**Keywords:** Femoral head necrosis, Tantalum rods, Total hip arthroplasty

## Abstract

**Background:**

Our study aimed to investigate the clinical outcomes and survival rates following porous tantalum rod surgery (PTRS) and conversion total hip arthroplasty (THA) subsequent to failed PTRS.

**Methods:**

A total of 38 subjects (40 hips) with osteonecrosis of the femoral head (ONFH) were included in this retrospective study between January 2008 and December 2011. All subjects were evaluated before surgery by using the Association Research Circulation Osseous (ARCO) classification system, the Japan Investigation Committee (JIC) classification and the Harris hip score (HHS). The endpoint of this study was set as final follow-up (including the survival time of PTRS and conversion THA). The rates of radiological progression were also evaluated. Patients who received conversion THA were further followed and compared to a control group of 58 patients with ONFH who underwent primary THA.

**Results:**

The mean follow-up time was 120.7 ± 9.2 (range, 104–143) months, and the overall survival rate was 75% at 96 months (ARCO stage II: 81.5%; stage III: 38.5%; JIC type C1: 83.3%; C2: 30%). The HHS before surgery was 59 (55–61), in contrast to 94 (91–96) at 96 months follow-up (*P* < 0.01). HHS in stage III show a significant poorer result compared to stage II at 24 months. HHS in Type C2 group show no significant difference compared to HHS before surgery at 24 and 60 months follow up (*P* = 0.91, *P* = 0.30). Twelve hips requiring secondary THA were followed for 66.9 ± 31.7 months, and control hips that underwent primary THA was followed for 75.4 ± 14.9 months. The HHS in the conversion group was 89 (86–93) and that in the primary THA group was 92 (79–95, *P* = 0.09) at the 5-year follow-up.

**Conclusion:**

In the mid-term follow-up, porous tantalum implants showed an encouraging survival rate in symptomatic patients in early stages (ARCO stage II) or with limited necrotic lesions (JIC type C1). In addition, our results did not demonstrated any difference between primary THA and conversion THA.

## Background

Osteonecrosis of the femoral head (ONFH) is considered a hip disorder characterized as collapsing of the femoral heads and secondary osteoarthritis [1]. ONFH is associated with a high morbidity rate in young patients, and an estimated 15,000–20,000 new patients per year in the USA alone are diagnosed with ONFH [2]. If left untreated, ONFH can evolve into articular incongruency and osteoarthritis [3]. Patients are more likely to require total hip arthroplasty (THA) once a signal of collapse occurs [4]. However, THA is not the best option for patients with ONFH in the early stages; therefore, if feasible, hip-preserving surgery is preferred [5]. Currently, hip-preserving surgeries, including core decompression, bone grafting, biocompatible implants and osteotomy, are variable and inconsistent [6–10].

Porous tantalum, which has become widely used in orthopedic surgeries, has been applied in clinical use for almost two decades [11]. This porous metal, which can provide high porosity, increased initial stability and good bone ingrowth qualities, is seeing wide use for primary and revision arthroplasties [12]. Porous tantalum rod surgery (PTRS) has the advantages of providing structural support and no need to consider donor-side complications [13]. Porous tantalum has a unique set of physical and mechanical properties and a high-volume porosity that allows rapid bone ingrowth. Conversion to THA is the the mainstay of treatment following tantalum rod failure [14–16]. Few studies have examined outcomes following failed joint-preserving techniques for THA. Davis et al. [17] reported that THA following a failed vascularized fibular graft was associated with significantly worse functional outcomes at 3 years compared with THA without a previous surgery. To our knowledge, no study in the literature has reported both the survival rate of patients who received tantalum implants at the mid-term follow-up and clinical outcomes following secondary THA compared with those following primary THA. The purposes of our study were as follows: a) to assess the hip survivorship of a group of patients with ONFH who were treated with PTRS; b) to evaluate the radiological progression and clinical outcomes by Harris hip score (HHS); c) to investigate whether the failure of PTRS would affect the clinical outcome of secondary THA.

## Methods

### Subjects undergoing PTRS

This study’s protocol was approved by the ethics committee of The First Affiliated Hospital of Guangzhou, University of Chinese Medicine. The inclusion criteria were as follows: a) radiographic criteria of ARCO stages II-III; and b) informed consent for participation in this study was provided. The exclusion criteria were as follows: a) radiographic criteria of ARCO stage IV; b) patients refused to accept PTRS as a hip-preserving surgery; c) active infectious disease in the affected hip; d) skin injury within the surgical area; e) secondary arthritis. Thirty-eight patients (40 consecutive hips, 25 males and 13 females) with nontraumatic ONFH were involved in this retrospective study. All the subjects had undergone core decompression combined with porous tantalum rod implantation (Trabecular Metal; Zimmer Trabecular Metal Technology, Allendale, New Jersey) between January 2008 and December 2011. All surgeries were performed by one surgeon (Wei H., MD). Subjects received an evaluation covering a preoperative medical history interview and an X-ray test in anteroposterior and frog leg lateral view, as well as MRI and CT scanning which is essential for ARCO stages and JIC classification. The ARCO classification, which has been updated several times since it was established in 1991, relies on multiple imaging results, such as MRI, CT and X-ray. In stage I, no significant changes are found in either X-ray or CT, and either MRI or scintigraphy is abnormal. Stage II is defined by changes in X-ray, such as sclerosis, but no crescent sign is present, and the subchondral bone is not affected. In stage III, the crescent sign and flattened femoral head are key features. Stage IV is defined by the presence of hip osteoarthritis [18]. The preoperative ARCO stage of the subjects was stage II in 27 hips and stage III in 13 hips [19]. The baseline characteristics of the patients (hips) who underwent PTRS are presented in Table 1.

**Table 1:** Baseline characteristics patients (hips) accepted PTRS

Charateristics	Parameter
Gender (female/male)	13/25
Sides affected (uni−/bilateral)	36/2
Etiology (no. of hips)	
Alcohol	9
Corticosteroid	26
Idiopathic	5
ARCO stage (hips)	
Stage II	27
Stage III	13
JIC type (hips)	
Type C1	30
Type C2	10

### PTRS technique

A 10-mm diameter porous tantalum rod was used for implantation. Each patient was placed in the supine position on a radiofluoroscopy table and prepared for surgery. First, a 3–5 cm lateral hip incision was made. The proximal lateral femur cortex was exposed when the iliotibial band and vastus lateralis were split. A core decompression technique was performed under fluoroscopic guidance. A 10-mm diameter core bone tunnel was developed by using a core reamer. The allograft bone was placed in the femoral head and impacted to support the femoral head. After the guide pin was measured, the porous tantalum rod was inserted under fluoroscopic guidance until the implant abutted the subchondral plate. Patients were routinely transferred to rehabilitation center and rehabilitation protocols were applied, including non-weight-bearing for the first 4–6 weeks in rehabilitation center and partial weight-bearing with a crutch for the following 6 weeks after discharged. All patients were allowed to bear full weight 12 weeks after the procedure. To evaluate the progression of collapse, we have evaluated the AP and frog-leg lateral view in plain radiographs images in PACS hospital imaging systems at each examination. We have modified the method from Nishii et al. [20]. First of all, we used a coin for calibration, and, on the serial plain radiographs, we drew and fit circles with the same radius to the femoral head contour. An increase distances between the maximum incursion into the femoral head and the fitted circles of the femoral head, as compared with the distances measured at the radiograph obtained 2 weeks after surgery, was defined as the amount of collapse. The greater distance of the AP and frog-leg lateral view was recorded at each follow-up. We defined radiographic progression when the amount of collapse was greater than 1 mm compared to the radiograph obtained 2 weeks after surgery.

### Conversion THA

THA was carried out when a subject suffered intolerable pain and limited functions or was dissatisfied with the outcome of PTRS. Routine THA (Synergy [Smith & Nephew, Memphis, Tennessee]) was performed after removing the tantalum rod from the original incision. However, it was usually impossible to remove the rod from a small incision, and a longer lateral incision and a hollow drill were needed to remove the implant successfully. To maintain the integrity of the tantalum rods and reduce the tantalum remains, instead of transecting the rods, a hollow drill was used to drill from the lateral cortical bone of the proximal femur to the end of the tantalum rod even though extra bone material was removed together with the tantalum rods. This procedure was used to minimize the remaining tantalum in the hip joint. The liner material used in all cases was highly cross-linked polyethylene.

### Subjects undergoing THA

Subjects undergoing revised THA were compared to a group of patients who received primary THA at late-stage ONFH. The patients in primary THA group were chosen according to the ratio of gender in conversion group. Generally, when one case in conversion THA group was included, another 4–6 cases with same gender, implants and close ages (±15) were chosen for further selection. Fifty-eight patients (58 hips) with nontraumatic ONFH who underwent THA (Synergy [Smith & Nephew, Memphis, Tennessee]) between January 2011 and December 2014 were included. The liner material used in all cases was highly cross-linked polyethylene. This control group included 26 females and 32 males, and the mean age was 42.7 ± 13.7 (range, 20–71) years. The inclusion criteria were as follows: a) diagnosed with symptomatic nontraumatic ONFH (at ARCO stage IV); and b) unwilling to undergo any kind of joint-preserving surgery and demand THA. The exclusion criteria were as follows: a) active infectious disease in the affected hip, b) skin injury within the surgical area, c) secondary arthritis. No patient was lost to follow-up.

### Statistics

Data parametricity was assessed using normality test. Parametric variables are reported as mean with standard deviation and were compared using student t test while nonparametric variables were reported as median with percentile 25 to percentile 75 and were compared using Mann Whitney U tests (conversion THA HHS vs primary THA HHS) or Wilcoxon signed-rank test (preoperative HHS vs postoperative HHS). Fisher exact test was performed to test associations between various parameters with survival. Kaplan-Meier survival analysis and log-rank tests were performed. All tests were two-sided. The results were considered significant at *P* < 0.05. Statistical analysis was performed using SPSS version 17.0 (SPSS Inc., USA), and graphs were generated by GraphPad Prism version 6.01 (GraphPad Software, USA).

## Results

### PTRS survival rate

**Fig. 1:**
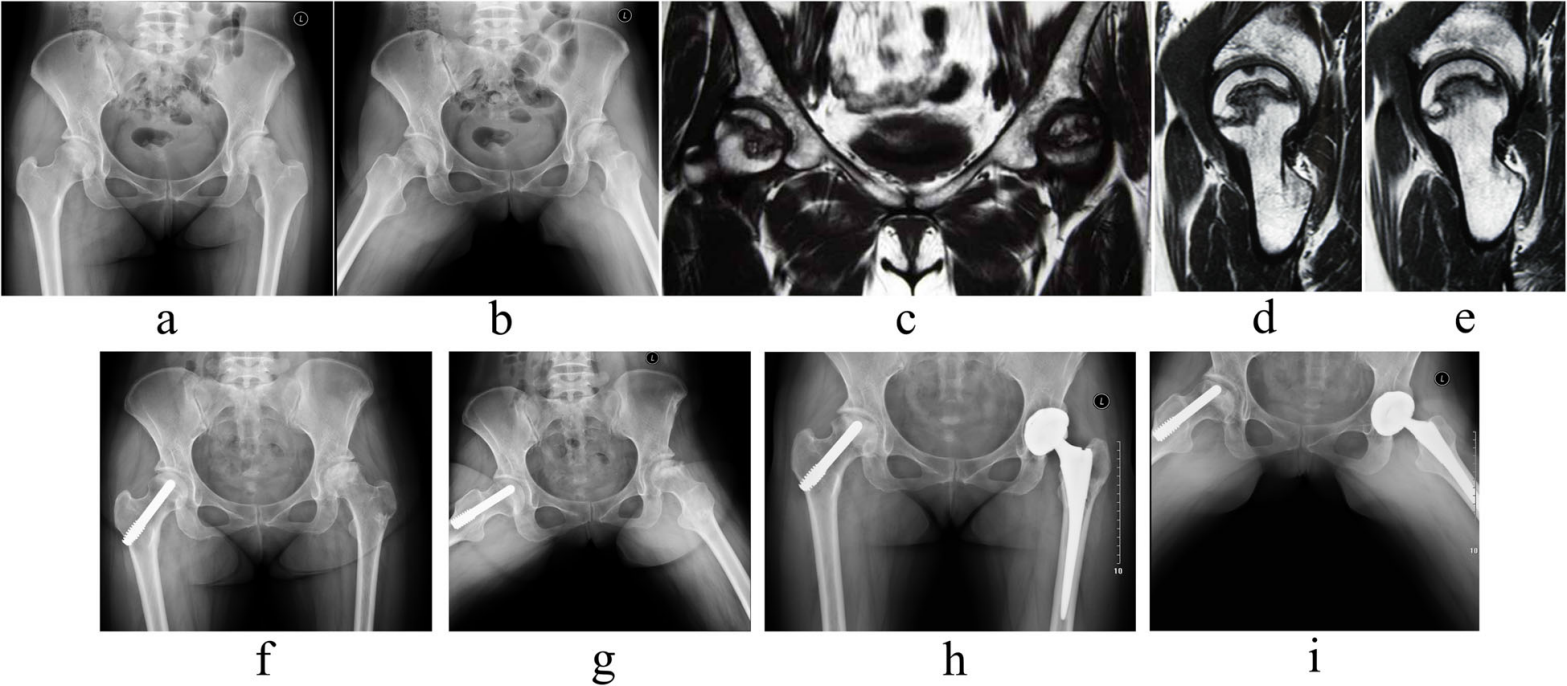
**a**, **b** Anteroposterior and frog lateral X-rays of bilateral hips showed that right hip was in ARCO stage II and JIC type C1. **c**, **d**, **e** preoperative MRI images showed right hip was involved serious ONFH but the lateral column was not involved. **f**, **g** the immediate radiographs followed by PTRS of right hip were shown. **h**, **i** even though mild subchondral bone collapse was observed, intact joint space was achieved with matched femoral head morphology at 9 years postoperatively, and the right hip was successfully preserved

**Fig. 2:**
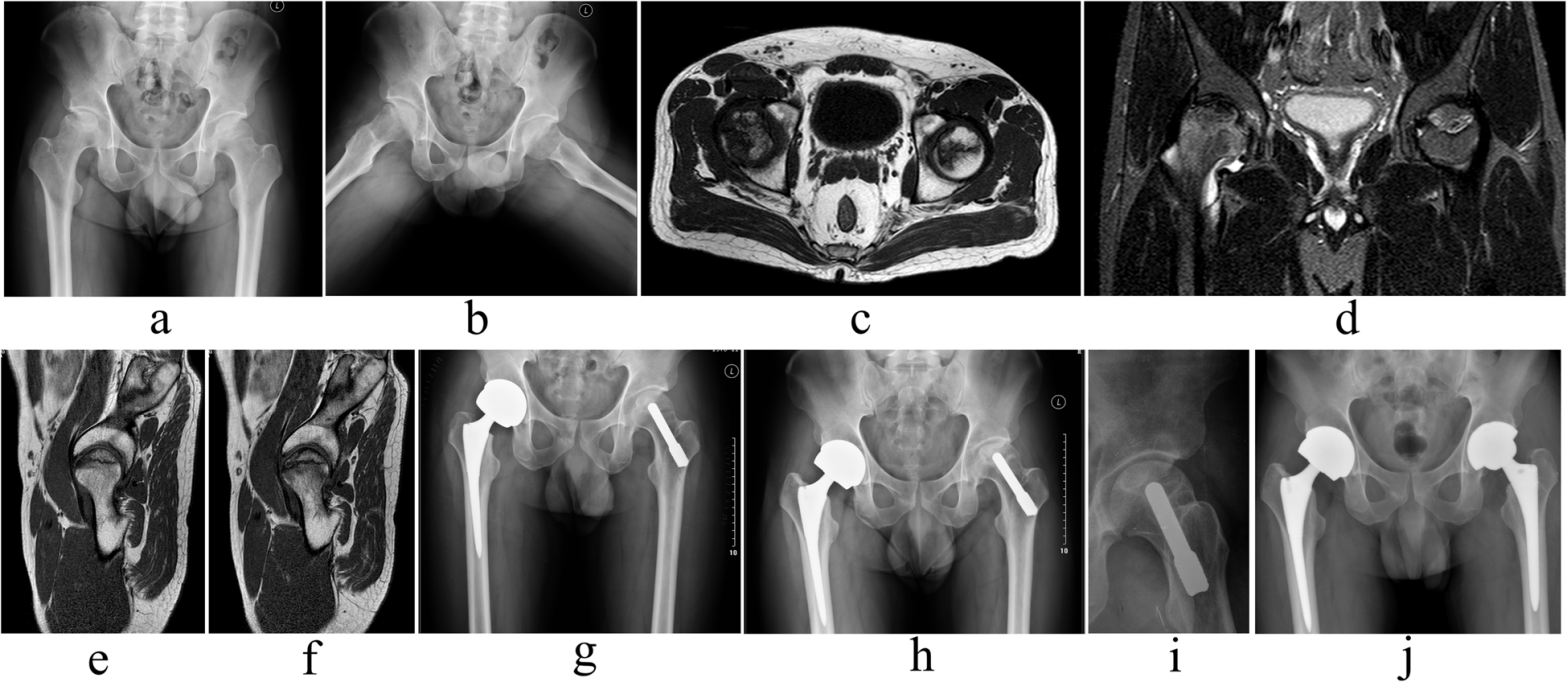
**a**, **b** Anteroposterior and frog lateral X-rays of bilateral hips showed that left hip was in ARCO stage II and JIC type C2. **c**, **d**, **e**, **f** preoperative MRI images showed left hip was involved serious ONFH, the lateral column was involved (sign of C2). **g** radiographs followed by PTRS of left hip was shown. **h**, **i** subchondral bone collapsing was observed with serious clinical symptom at 2 years postoperatively. **j** THA was performed

No clinical complications were noted after the operation. At an average of 54.4 ± 32.8 months (13–99) after surgery, 12 hips (30%) required conversion to THA. Persistent pain and joint destruction were the indications for THA (Figs. 1 and 2). The total mean follow-up time (including PTRS survival time and conversion THA follow-up time) was 120.7 ± 9.2 (range, 104–143). The mean PTRS follow-up time was 100.1 ± 4.1 (13–143) months. A total of 37 hips were followed for a period of ≥24 months, and 3 hips failed in the first 24 months. The survival rate was 92.5% (37/40) after 24 months. Thirty-three hips were followed for ≥60 months, and 7 hips were converted to THA. The survival rate was 82.5% (33/40) after 60 months. Thirty hips were successfully followed for ≥96 months, and 10 hips failed in the first 96 months. The survival rate was 75% (30/40) after 96 months. The survival rates of ARCO stages or JIC types at particular time points were show in Table.2. Sex (*p* = 0.72), etiology (*p* = 0.86), age (*p* = 1.00), and the preoperative HHS (*p* = 0.91) did not show significant differences in the survival rate (Table 2).

**Table 2:** Survival rate in different groups

Parameters	Fails / Survives	Survival rate %	*P value*
ARCO stages (n. of hips)			
At 24 months			*P* = 1.00
II	2/25	92.6%	
III	1/12	92.3%	
At 60 months			*P* = 0.45
II	4/23	88.9%	
III	4/9	69.2%	
At 96 months			***P = 0.04***
II	4/23	81.5%	
III	6/7	53.8%	
JIC types (n. of hips)			
At 24 months			*P* = 1.00
C1	2/28	81.5%	
C2	0/10	100%	
At 60 months			*P = 0.34*
C1	4/26	86.7%	
C2	3/7	70.0%	
At 96 months			***P = 0.03***
C1	5/25	83.3%	
C2	5/5	50.0%	

### Hip scores

Hip scores were presented as median (percentile25-percentile75). The postoperative HHS within the survivor group at the 96 months follow-up was 94 (91–96), while the preoperative HHS was 59 (55–61, *p* < 0.01). The HHS of different ARCO stages and JIC types at particular time points were show in Table.3.

**Table 3:** HHS before surgery and follow-up

Time points	Groups (HHS Median [percentile25-percentile75])				Overall
	Stage II	Stage III	Type C1	Type C2	
Pre-op	59 (56–61)	53 (46–56)	57 (52–61)	61 (55–63)	59 (55–61)
24 ms	86 (83–90) ^**^	79 (68–84) ^**, ¶¶^	86 (82–90) ^**^	68 (61–83) ^¶¶^	85 (79–90) ^**^
60 ms	89 (88–91) ^**^	90 (78–91) ^**^	90 (87–91) ^**^	82 (65–88) ^¶^	89 (86–91) ^**^
96 ms	94 (90–96) ^**^	92 (90–95) ^**^	94 (91–96) ^**^	89 (70–94) ^*^	94 (91–96) ^**^

^**^: Significant difference compared to pre-op HHS score in each group (*P* < 0.01); *: Significant difference compared to pre-op HHS score in each group (*P* < 0.05)

^¶^: Significant difference compared to stage II / Type C1 at the same time point (*P* < 0.05)

^¶¶^: Significant difference compared to stage II / Type C1 at the same time point (*P* < 0.01)

### Radiological progression

The overall rate of radiological progression was 55.0% (22/40) (12 failed hips and 10 survival hips). All hips that underwent conversion THA exhibited serious radiological progression. The radiological progression rate was 48.1% (13/27) in stage II, 69.2% (9/13) in stage III, 46.7% (14/30) in type C1 and 80% (8/10) in type C2. Hips in stage III did not show a significantly higher progressive rate than hips in stage II (*p* = 0.39). However, the survival rate in stage III progressive hips was only 11.1% (1/9), which was much lower than the survival rate in stage II progressive hips (76.9%, 10/13) (*p* = 0.02). Although the ratio of progressive hips in type C2 was much higher than that in type C1, the difference was not statistically significant (*p* = 0.08). The survival rate in type C2 progressive hips was 12.5% (1/8), which was significantly lower than the survival rate in type C1 progressive hips (71.4%, 10/14) (*p* = 0.02).

### Revised patients and the control group

The demographic data consisted of the number of patients (hips), age (at the time of surgery), sex, and etiologies and are presented in Table 4. The HHS in the conversion group before conversion THA was 56 (50–59). After secondary THA, the HHS significantly increased to 88 (85–93, *p* < 0.01) at 60 months follow up. One patient in the control group needed revision THA because of infection. Nine hips in the conversion group and 57 hips in the control group were followed for 36 months, and no difference in the HHS was found (*p* = 0.31). Seven hips in the conversion group and 54 hips in the control group were followed for 60 months. No difference in the HHS was found (*p* = 0.09, Table.4).

**Table 4:** Comparison of baseline and clinical outcome of conversion THA group and primary THA group

Characteristics	Conversion THA	Primary THA	*p*
No. of hips	12	57	*–*
Age (year, mean ± SD, [range])	44.0 ± 10.0 (31–63)	42.7 ± 13.7 (20–71)	*p = 0.97*
Gender (female/male)	4/8	25/32	*p = 0.78*
Follow-up time (year, mean ± SD, [range])	66.9 ± 31.7	75.4 ± 14.9	*p = 0.15*
Etiology			*p = 0.42*
Alcohol	3	13	
Corticosteriod	8	37	
Idiopathic	1	17	
ARCO stage			*p = 0.71*
Stage III	2	15	
Stage IV	10	42	
JIC classification			*p = 0.51*
Type C1	5	18	
Type C2	7	39	
HHS (Median (P25-P75))			
Pre-op	56 (50–59)	56 (42–66)	*p = 0.41*
36 months Pos-op	91 (87–94)	94 (85–98)	*p = 0.31*
60 months Pos-op	88 (85–93)	92 (88–96)	*p = 0.09*

## Discussion

In our study, we reported survival rates of 92.5% at 24 months, 82.5% (33/40) at 60 months, 75% at 96 months follow-up. A significant difference in survival rates was found between patients with stage II and stage III disease at 96 month. Hips in stage II had a higher survival rate than those in stage III. According to the JIC classification, hips in type C2 had the highest failure rate (50%), while those in type C1 had a failure rate of 28.6%. In cases of serious ONFH, PTRS was associated with high failure rates. Results of HHS in stage III was significantly poorer than that in stage II at 24 months, but similar at 60 and 96 months follow up. HHS results in Type C2 were statistically lower compare to Type C1 at 24 and 60 months. Moreover, PTRS did not improve the HHS in Type C2 group compared to HHS before surgery. Interestingly, the improvement of HHS in stage III was significantly greater at 60 and 96 months follow up compared to stage II hips. However, similar results were not show between Type C1 and C2. Since the postoperative scores between stage II and stage III were similar, the greater improvement in HHS observed in stage III appears to be due to the worse preoperative HHS scores, rather than better superior final outcome. Second, radiological progression existed in more than half of our hips, but the failure rate varied widely according to stage and type. Hips in stage III or type C2 had a higher failure rate when the disease progressed. Finally, THA can significantly ameliorate clinical function when tantalum rod insertion fails. Instead of radiographic progression, the endpoint of the PTRS was THA because mild subchondral bone collapse might not predict unsatisfactory clinical outcomes. The survival rates in radiological progressive stage II and type C1 hips were much higher than those in stage III or type C2. This finding might suggest that radiological progression is an important sign of failure in cases of severe ONFH and could be explained as limited collapse may not affect the integrity of cartilage. Porous tantalum rods can provide temporary stabilization inside the femoral head and extra time for bone repair. The necrotic bone is slowly replaced by new bone, and during this period, a slight collapse may be observed. A porous tantalum rod is not a permanent solution for preventing collapse but can provide additional time for increasing stabilization.

Whether patients with ARCO stage III (or stage III or VI in Steinberg stages) should receive tantalum rods implantation was controversial in the early years. Varitimidis have reported a success rate of 75% at 6 years follow up in patients with Steinberg stage III or VI [16]. However, a recent study from Ma et al. report only 36.0% in 5 years [21]. Collapsing of the femoral head is a key point in ONFH. Hip conservation procedures become less effective when femoral head have collapsed and THA remains the common option [15]. Our finding reports a significant poorer success rate in ARCO stage III patient, confirming the effect of a porous tantalum implant in treating post-collapse femoral head is not ideal, which is in agreement with most of other centers.

Success rate in early stage ONFH show encouraging compared to Floerkemeier’s research [22] (all cases are in early stage but reporting 44% in 529 days follow up), one of the possible reason is that the prolonged non-weight-bearing rehabilitation procedure for all patients received PTRS. Meanwhile, we should not neglect that the rehabilitation procedure for the PTRS is much longer than patient received THA. THA patients can mobilize and get back to daily life without extra time and cost for rehabilitation. In contrast, PTRS patients have to cost a lot and spend more time (at least 12 week for non-full weight-bearing). The cost for rehabilitation should be further studied in our future works.

Few studies have reported the clinical outcome of ONFH receiving PTRS according to different JIC types. Because the cases were those treat during 2008–2010 and we had few clinical reports for consultation, ONFH with type C2 were also recruited to treat with PTRS. According to our results, patients with type C2 are more likely to fail and conversion THA is prone to occur in patients with JIC type C2. Patients with large size of necrosis should not accept PTRS because of the low success rate for joint-preserving.

A total of 12 failed tantalum rod implants needed secondary surgery in our series of 40 patients at our institution. Our results did not demonstrate any difference between primary THA and conversion THA. The clinical function of THA in patients with ONFH is potentially compromised by failed tantalum rod implantation and subsequent removal, but due to the small sample size and limited follow-up time of this study, the negative effect was not obvious.

The progression of articular surface collapsing and preservation of the femoral head in ONFH have always been complex issues. Unlike Veilette’s study [23], the survival rate among different pathogeneses and between patients with or without chronic disease did not show significant differences. The HHS at each stage and classification was significantly improved in survival hips. The efficiency of PTRS reported in previous studies is highly inconsistent.

Survival rates for hips receiving PTRS or a similar technique in existing studies vary widely. However, the overall survival rates may be based on the severity of the case. The highest survival rate reported in Liu et al’s study was 83.7%: they recruited 49 hips, but only 2 hips were in Steinberg stage IIIA and were followed for 15.2 months. The high proportion of early-stage cases and short follow-up time might not tell the whole picture. Our results were similar to those from Varitimidis et al., who reported a survival rate of 70% after a 6-year follow-up, and their cases consisted of 9 hips in Steinberg stage I, 7 hips in stage II and 10 hips in stage III. Several studies reported a much lower survival rate than ours. Nadeau et al. and Ma et al. reported survival rates of only 42.8 and 52.9%, respectively, in less than 4 years of follow-up. More than half of their included cases were in an advanced stage (higher than stage III). However, higher survival rates were reported when PTRS was combined with techniques such as vascularized bone grafts. Nevertheless, vascularized bone grafts are associated with potential risks, such as an extensive surgical procedure, donor site complications, and surgical infection of an extra incision. The endpoint of all the studies mentioned above was THA (Table 5).

**Table 5:** Literature review of tantalum rod

	Hips	Design	Patient selection (hips)	Follow-up (Mons)	Survivorship	End point
Tsao et al. (2005) [24]	94	Prospective	Steinberg I (1) II(93)	48	72.5% at 48mons	THA
Veilette et al. (2006) [23]	58	Retropective	Steinberg I (1) II(49) III (8) (1)II(93)StageI(1)II(49)l(8)	24	68.1% at 48mons	THA
Nadeau et al. (2007) [13]	18	Prospective	Steinberg III (3) IV(15)	23.2	42.5% at 48mons	THA
Varitimidis et al. (2009) [16]	26	Prospective	Steinberg I (9) II(7) III (10)	38	70% at 6 years	THA
Liu et al. (2010) [25]	49	Prospective	Steinberg I (21) II (26) IIIA(2)	15.2	83.7%	THA
Floerkemeier et al. (2011) [22]	23	Retropective	ARCO I II	529(days)	44%	THA
Liu et a al. (2013) [26]	94	Prospective	Steinberg I (27) II (49) III(18)	37.3	I 88.9% II 79.6% III 55.6%	THA
Ma et al. (2016) [27]	104	Retrospective	ARCO II(42) III(62)	42	52.9%	THA
Hu et al. (2018) [28]	29	Prospective	ARCO I (5) II(24)	5.4 (year)	93.1%	THA

The limitations of our study were as follows: a) its retrospective nature; b) there was a small number of hips and no control group treated without porous tantalum rods or treated nonoperatively; c) although we used a mid-term follow-up of 8 years for both survival and failure analyses, it was obviously not long enough because ONFH affects young patients; d) patients in the conversion THA group were not formally matched neither by age and gender nor ARCO stages and JIC types. More completely matched cases are needed to determine the potential effect of the preoperative status; e) Insufficiency numbers of cases in our study might underpower the difference between groups. For example, there were more than 20% higher in the rate of radiological progression between in stage III compared to stage II, but the difference was not statistical. A similar situation was observed in Type C2 compared to C1 (more than 30% higher rate of progression but no statistical difference). In order to avoid this Type II error, more cases are need and a multiple centers study is our future study, and f) with the limited condition we have, we were not able to evaluate the rate of linear wear in both conversion THA and primary THA group.

## Conclusions

In summary, PTRS combined with core depression, necrotic tissue removal, and allograft bone grafting for ONFH showed an encouraging survival rate in symptomatic patients with ONFH in the early stages or with limited necrotic lesions but not in patients with collapsed femoral heads or large necrotic lesions. Patients with necrotic areas involving large areas or in late-stage THA are not suitable for tantalum rod implantation. In addition, our results did not demonstrated any difference between primary THA and conversion THA. Finally, studies with larger revision cases and longer follow-up times are needed to evaluate the potential effect of failed tantalum rods.

## Data Availability

The datasets used and/or analyzed during the current study are not publicly available due to feasibility but are available from the corresponding author on reasonable request.

## Abbreviations

PTRS: Porous Tantalum Rods Surgery
ARCO: Association Research Circulation Osseous
JIC: Japanese Institute Committee
HHS: Harris Hip Score
ONFH: Osteonecrosis of the Femoral Head
THA: Total Hip Arthroplasty
